# Thermoresponsive Nanogels Based on Different Polymeric Moieties for Biomedical Applications

**DOI:** 10.3390/gels6030020

**Published:** 2020-07-04

**Authors:** Sobhan Ghaeini-Hesaroeiye, Hossein Razmi Bagtash, Soheil Boddohi, Ebrahim Vasheghani-Farahani, Esmaiel Jabbari

**Affiliations:** 1 Biomedical Engineering Department, Faculty of Chemical Engineering, Tarbiat Modares University, Tehran 14115, Iran; so.ghaeni@gmail.com (S.G.-H.); hosseinrazmi1992924@gmail.com (H.R.B.); 2 Biomimetic Materials and Tissue Engineering Laboratory, Department of Chemical Engineering, University of South Carolina, Columbia, SC 29208, USA; jabbari@cec.sc.edu

**Keywords:** nanogels, thermoresponsive, drug delivery

## Abstract

Nanogels, or nanostructured hydrogels, are one of the most interesting materials in biomedical engineering. Nanogels are widely used in medical applications, such as in cancer therapy, targeted delivery of proteins, genes and DNAs, and scaffolds in tissue regeneration. One salient feature of nanogels is their tunable responsiveness to external stimuli. In this review, thermosensitive nanogels are discussed, with a focus on moieties in their chemical structure which are responsible for thermosensitivity. These thermosensitive moieties can be classified into four groups, namely, polymers bearing amide groups, ether groups, vinyl ether groups and hydrophilic polymers bearing hydrophobic groups. These novel thermoresponsive nanogels provide effective drug delivery systems and tissue regeneration constructs for treating patients in many clinical applications, such as targeted, sustained and controlled release.

## 1. Introduction

### 1.1. Hydrogels

**Figure 1:**
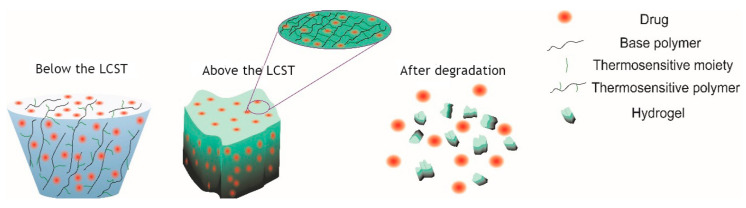
Schematic degradation of thermosensitive hydrogel below and above LCST.

Hydrogels are three-dimensional cross-linked structures based on natural or synthetic polymers. Hydrogels can be produced in different physical forms, such as slabs, macroparticles, nanoparticles and films [1,2]. Promising properties, such as high water content, biocompatibility and degradability, make hydrogels very useful for biomedical applications [3,4,5]. A salient example of biocompatible hydrogels is the injectable and temperature-sensitive poly(amino carbonate urethane) (PACU) hydrogel, which has been used as a delivery vehicle for sustained release of human growth hormone factor [6]. Similarly, the alternating hydrophilic/hydrophobic properties of poly(N-isopropylacrylamide)-co-methacrylate [P(NIPAM-co-MA] hydrogel are used for temperature sensing in biomedical applications [7]. Due to nanogels’ structural properties, hydrogels are abundantly used in drug delivery systems and fabrication of tissue scaffolds [6,8,9]. The structure of hydrogels can be modified by conjugation with appropriate ligands to improve properties such as drug entrapment, release profile and targeting [10,11,12]. In a recent study, Liao et al. presented a novel method for the preparation of multi-responsive DNA-acrylamide (DNA-AAM)-based hydrogel microcapsules [13]. Hydrogels are also used extensively in tissue engineering as more suitable materials to fabricate biodegradable scaffolds for tissue regeneration [14,15]. One important parameter which can affect the biodegradability of hydrogels is the lower critical solution temperature (LCST). As a definition, when two immiscible liquid phases appear as a result of temperature increase at the different compositions of polymer and solvent, the minimum of the coexistence curve of the phase diagram is the LCST [16]. Figure 1 represents the schematic degradation mechanism of a thermoresponsive hydrogel for drug delivery applications, which is controlled by the LCST. Below the LCST, the thermosensitive hydrogel is in the solution state but when temperature increases and it is higher than LCST, gelation occurs, and for in-vivo or in-vitro conditions under specific circumstances such as exposing to enzymes, hydrogel can degrade. Hydrogels also have shortcomings, which leads to uncertainty in their applications in medicine, which can be addressed by transforming their macro- and micro-structure to the nanoscale.

### 1.2. Nanostructured Materials

Nanostructured devices are a novel class of materials with many biomedical applications [17,18,19]. The preeminent property of nanostructures is their high surface-to-volume ratio, which makes these structures injectable and improves their penetration between physiological barriers in the human body. These structures enhance disease treatment with minimum side effects and toxicity [20]. Nanostructures can be prepared in different forms, like nano-films [21], nanofibers [22,23], nanoparticles [24,25], and nanogels [26], which have the potential to be used in both drug delivery systems and tissue engineering. Recent studies indicate that nanoscale structures impact biological response; thus, they can be used to modify the surface of medical implants to decrease undesired biological responses [27,28]. Bamberger et al. synthesized a polysaccharide-based nanostructure within the 100–200 nm size range, which was modified with dextran (Dex) and polyethylene glycol (PEG) and assessed for its ability to bind to immune cells. They reported that the surface modification of nanoparticles with dextran (DEXylation) enhanced targeting with a desirable immune response [29].

**Figure 2:**
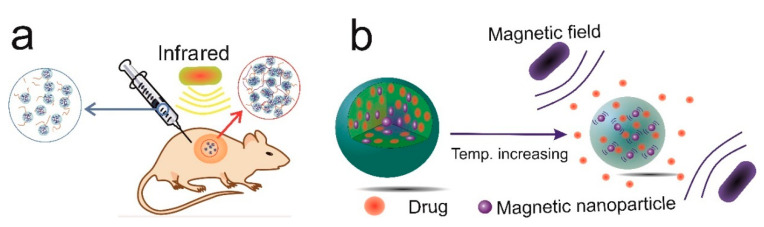
(**a**) Infrared-responsive nanocarriers for in-situ forming hydrogels as a drug delivery system and tissue scaffold and (**b**) magnetic fields and temperature-responsive nanocarriers for increasing drug release.

In drug delivery systems, nanostructures can be injected subcutaneously or intravenously to deliver the loaded drugs to the site of injury or disease with minimum cell toxicity and immune response [30]. The surface of the drug delivery system can be modified with ligands that can be detected by receptors on the surface of malignant tumours in cancer therapy [31,32,33,34]. Stimuli-responsive nanostructures have the potential to be used for targeted delivery and controlled drug release. The pH [35], temperature [36], magnetic field [37] and redox reaction [38,39] are the most commonly used environmental factors in stimuli-responsive systems. Stimuli-responsive nanocarriers have the potential to induce enhanced permeability [40,41]. In addition, targeted delivery enables selective delivery of the drug to the diseased tissue while leaving the healthy tissue unharmed [42]. Despite the numerous advantages of nanocarriers for drug delivery, there are some challenges to be tackled, including difficulty of synthesis, low stability, and the circulation time of nanocarriers in blood circulation. In some cases, toxicity to normal cells and non-biodegradability are the main deficiencies of these structures [43]. Figure 2 shows two general types of modified nanocarriers, which can be used for the targeted delivery of both hydrophilic and hydrophobic drugs.

## 2. Nanogels

**Figure 3:**
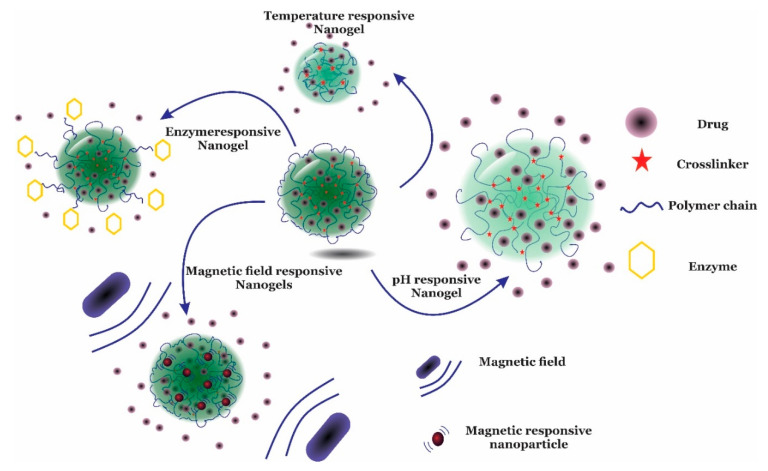
Scheme of different stimuli-responsive nanogels in response to temperature, enzyme, the magnetic field, and pH in drug delivery applications.

Nanogels are three-dimensional (3D) structures that are able to swell several times their non-swollen form [44,45]. Nanogels are nanostructured hydrogels with the advantages of both nanostructured materials and hydrogels. The two main characteristics of nanogels are their small size (up to 1000 nm) and high swelling ratio or water content [46]. Due to these properties, nanogels have become an excellent platform in many medical applications, including photo-imaging [47], tissue regeneration [48], cancer therapy [49] and gene delivery [50]. This is based on their remarkable characteristics, such as their high capacity for drug entrapment and release [51], tailorable size [52], tuneable toxicity [53], high stability, controlled and sustained drug release [54], precise targeted delivery [55], and high biodegradability [56]. Nanogels can be used for drug delivery through oral [57], pulmonary [58], nasal [59], intra-ocular [60] and topical [60] pathways. There are many methods that can be used to prepare stimuli-responsive nanogels for targeted delivery. Thermosensitive [61], pH-sensitive [62,63], glucose-sensitive [64], redox-sensitive [65], and magnetic-field-sensitive [66] nanogels are applicable to the treatment of many diseases (Figure 3). Furthermore, nanogels can be tailored as dual or multi-responsive structures [42,67]. Deng et al. explored the synthesis and properties of poly (N,N-dimethyl aminoethyl methacrylate -g- Ethylene glycol) P(DMAEMA-g-EG) nanogel carriers with 190–600 nm diameters, which showed pH, ionic strength and temperature sensitivity with LCSTs of about 35 °C [68]. The objective of this review is to describe the properties of thermoresponsive nanogels, with a focus on polymeric moieties that influence the thermoresponsive behavior of nanogels in biomedical applications. Given the wide range of applications, thermoresponsive nanogels are promising for many medical uses, such as for sensors, imaging, diagnosis, treatment and gene delivery. Different types of thermosensitive generator side groups, followed by various applications of the thermoresponsive nanogels, will be presented.

### Thermosensitive Nanogels

Thermosensitive nanogels are soft nanostructured materials that respond to temperature changes in the surrounding medium. Two approaches are used to prepare thermosensitive nanogels. In the first approach, thermosensitive polymer units are incorporated in the backbone or the main structure of a nanogel-forming polymer to induce thermosensitivity. In the second approach, hydrophobic moieties are attached as side groups to a hydrophilic polymer backbone to impart temperature sensitivity [69,70,71]. The LCST of the polymer decreases as the fraction of polymer units with a hydrophobic side group is increased. Therefore, the gelation temperature can be tuned by changing the degree of substitution of backbone units with hydrophobic moieties.

The mechanism responsible for thermosensitivity is the extent of the molecular interactions which could be categorized as hydrophobic or hydrophilic, depending on the free energy change of the surrounding solvent. A positive change or a negative change in the free energy of a mixture indicates its hydrophobicity or hydrophilicity, respectively [72]. Association of water molecules, as the governing interaction in the system, is the main cause of free energy change. Water molecules at temperatures lower than the LCST align well around hydrophilic parts. However, at temperatures above the LCST, owing to the hydrophobicity of the surrounding groups, water molecules start detaching, with a consequent phase separation between the water and polymers. Moreover, as the temperature increases, the alignment of water molecules collapses due to hydrophobic moieties, and the entropy of the system increases, which leads to gel formation. In other words, when the temperature is lower than the LCST, hydrophilic–hydrophilic interactions are stronger than hydrophobic–hydrophobic interactions, and this increases polymer solubility. As the temperature increases, hydrophobic–hydrophobic interactions become more important than hydrophilic–hydrophilic interactions, which eventually leads to aggregation of hydrophobic moieties and nanogel formation.

## 3. Thermosensitive Polymers

### 3.1. Polymers Bearing Amide Groups

#### 3.1.1. PNIPAM

**Figure 4:**
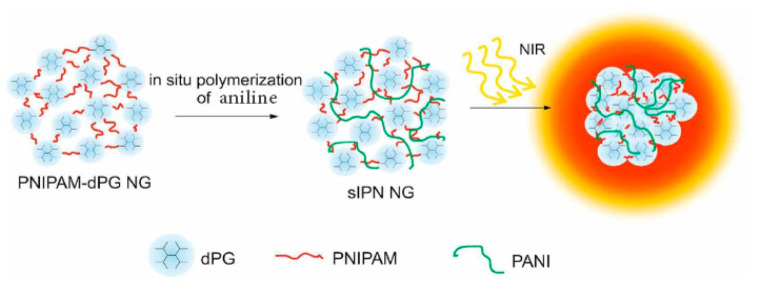
In-situ mechanism for near-infrared absorbing nanogels based on PNIPAM-dPG-PANI for photothermal cancer therapy. Adapted from Ref. [79] reproduced by permission of The Royal Society of Chemistry.

Poly (N-isopropyl acrylamide) (PNIPAM) is a thermosensitive polymer containing hydrophilic (C = O, NH) and hydrophobic groups (i.e., CH_3_). PNIPAM is synthesized by free radical polymerization. This polymer is abundantly investigated in tissue engineering and drug delivery applications [73,74,75]. Although the LCST of PNIPAM is about 32 °C, which makes this polymer an appropriate temperature-sensitive biomaterial, the non-biodegradability of PNIPAM impedes its widespread use in clinical applications. PNIPAM-based drug carriers can be modified with different functional groups for targeted drug delivery [76], controlled release [77], imaging and tracking [47], as well as other functionalities [66]. Zhou et al. investigated doxorubicin (DOX) release from a temperature-sensitive and photoluminescent hydrogel using PNIPAM and cadmium telluride quantum dots (CdTe QDs) (photoluminescent inducer) with polyacrylamide (PAA) as a crosslinker. Results demonstrated that the rate of drug release could be adjusted by external temperature [78]. Molina et al. formulated a near-infrared (NIR) absorbing nanogel based on N-isopropylacrylamide– dendritic polyglycerol–polyaniline (NIPAM-dPG-PANI) for photothermal cancer therapy (Figure 4). The size of nanogels was about 150–240 nm, and in-vitro MTT [3-(4,5-Dimethylthiazol-2-yl)-2,5-Diphenyltetrazolium Bromide] assays on A2780 cells and in-vivo (mice) investigations were performed. In this research, the results indicated that mice could tolerate a 500 mg/kg dose of nanogels in 5 days without substantial toxicity [79]. Śliwa et al. synthesized a temperature-responsive nanogel with a hydrodynamic diameter of 150–650 nm for the controlled release of orange II. This nanogel was prepared by polymerization of 1-vinylimidazole (Vim) and PNIPAM monomers, with bisacrylamide (BAM) as the crosslinker [51].

#### 3.1.2. PNIPMAM

**Figure 5:**
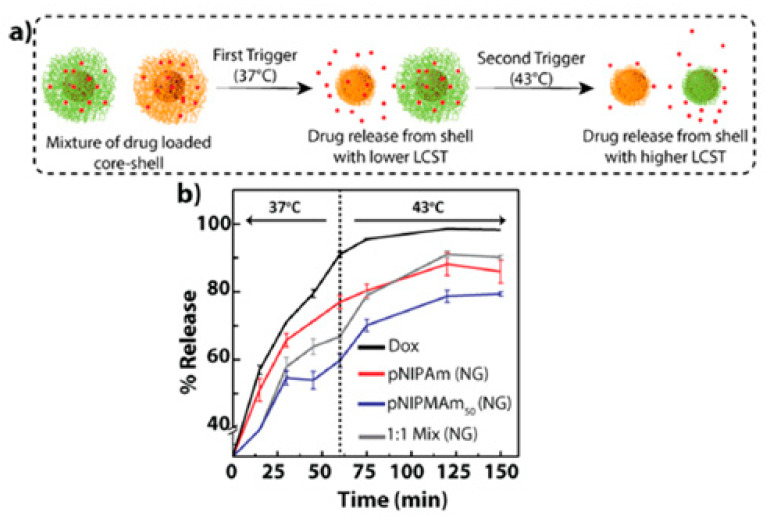
NIPAM and NIPMAM-based thermosensitive core–shell nanogels for triggered and sustained release of DOX. (**a**) Schematic diagram showing the trigger-based release and (**b**) sustained release of DOX from the nanogels. Adopted from Ref. [84] reproduced by permission of American Chemical Society. Further permissions related to the material excerpted should be directed to the ACS.

Poly (N-isopropyl methacrylamide) (PNIPMAM) is another amide-bearing thermosensitive polymer that contains methyl groups attached to the α-carbon, with a higher swelling ratio in the aqueous medium compared to PNIPAM [80]. Despite having similar characteristics to PNIPAM, there are remarkable differences between the two polymers for drug delivery applications. One important difference is the higher LCST of PNIPMAM (38 °C) compared to PNIPAM (32 °C), due to the higher hydrophilicity of PNIPAM compared to PNIPMAM [81]. Cors et al. synthesized a core–shell thermosensitive nanogel based on PNIPMAM as the core and PNIPAM as the shell in order to understand the swelling and shrinking behaviour of the polymer [82]. The results of this study indicated a linear increase in swelling with temperature in the range of 25 to 35 °C. Peters et al. prepared a thermosensitive PNIPMAM-based core–shell nanogel for cancer therapy for controlled and triggered release of DOX. The cytotoxicity of the synthesized nanogels was investigated with L929 fibroblasts, and low toxicity on cells was demonstrated [83]. In Figure 5, Deshpande et al. prepared core–shell nanogels using PNIPMAM as the shell and gold nanoparticles as the core for sustained, triggered release of DOX [84].

#### 3.1.3. PDEAAM 

Poly (N,N-diethylacrylamide) (PDEAAM) is a thermosensitive polymer studied by Idziak et al. to determine the LCST of PDEAAM at different concentrations of sodium dodecyl sulphate (SDS), using ultraviolet (UV) spectroscopy and differential scanning calorimetry (DSC). They showed that PDEAAM had a sharp phase transition with an LCST of about 33 °C [85]. Different research groups have studied PDEAAM’s properties, such as enhancement of thermosensitivity by copolymerization with 2-hydroxyethyl methacrylate (HEMA) [86] and thermal responsivity of PDEAAM hydrogels prepared by γ-ray irradiation [87]. In the past decade, PDEAAM has been used for the preparation of responsive hydrogels [88,89,90], micelles [91,92] and nanogels [93,94] for biomedical applications. Lu et al. prepared non-ionic and thermosensitive nanogels with 100-nm diameters, based on N,N-diethyl acrylamide (DEA) and N,N-dimethylacrylamide (DMA), which can be used for DNA separation by microchip electrophoresis [95]. Rieger et al. synthesized thermoresponsive PEGylated micelles and nanogels to form core–shell nanostructures, with diameters of 800 and 550 nm at 15 and 70 °C, respectively [96], which could be used as drug carriers.

#### 3.1.4. PVCL

Poly (N-vinyl caprolactam) (PVCL) is a water-soluble amphiphilic polymer, with non-ionic, thermoresponsive characteristics. Polymer molecular weight can affect LCST. Because of its biocompatibility and low cytotoxicity, PVCL is considered an ideal thermoresponsive polymer for biomedical applications [97,98,99], particularly when compared to PNIPAM [100,101,102]. PVCL conjugation with hydrophilic units such as PEG or derivatives of PEG enables the synthesis of temperature-responsive block copolymers. For temperatures above the CPT of PVCL, these block copolymers act as amphiphilic structures to form well-defined nanoscale aggregates by self-assembly pathways. Such stimuli-responsive structures, due to their ability to assemble or disassemble without using any additive, have immense potential for use in advanced drug delivery systems [103].

**Figure 6:**
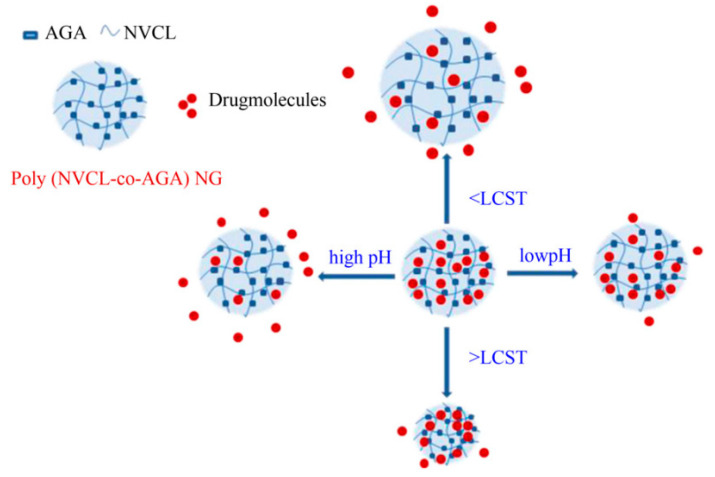
Dual-responsive nanogels based on N-vinyl caprolactam (VCL), which illustrate an increase in drug release at high pH, and the polymer network structure is collapsed at high temperature resulting in lower drug release. Adapted from Ref. [105] reproduced by permission of Elsevier.

PVCL has also been utilized for the preparation of stimuli-responsive nanoparticles and nanofibers [37]. For instance, González et al. prepared thermoresponsive nanofibers via the electrospinning technique and investigated their use in drug delivery systems. PVCL and hydroxymethyl acrylamide were copolymerized and used to generate Rhodamine B (RhB)-loaded nanofibers with diameters in the range of 550–1200 nm. The results demonstrated that the copolymer could be used as a biosensor or as a matrix for controlled drug delivery [22]. In another study, Kehren et al. prepared polycaprolactone (PCL) microfibers modified with PVCL-based nanogel and investigated water uptake and degradability. The results demonstrated that the thermosensitivity of nanogels was preserved irrespective of whether the nanogels were in or out of the microfiber surface. Additionally, the PVCL nanogels in the structure regulated the degradability of the PVC-modified PVCL nanogels [104]. Madhusudana et al. synthesized dual-responsive nanogels by copolymerization of N-vinyl caprolactam (VCL) and acrylamidoglycolic acid (AGA) for applications in cancer therapy. The in-vitro release of the anticancer drug 5-fluorouracil (5-Fu) from VCL-AGA nanogels was influenced by both pH and temperature [105] (Figure 6).

Other previously studied thermosensitive nanogels with polymers bearing amide groups are also introduced in Table 1. Parameters such as size, interactions, thermosensitive part, therapeutic agent, and application are provided for better comparison.

### 3.2. Polymers Bearing Polyether Groups

#### PEG

**Figure 7:**
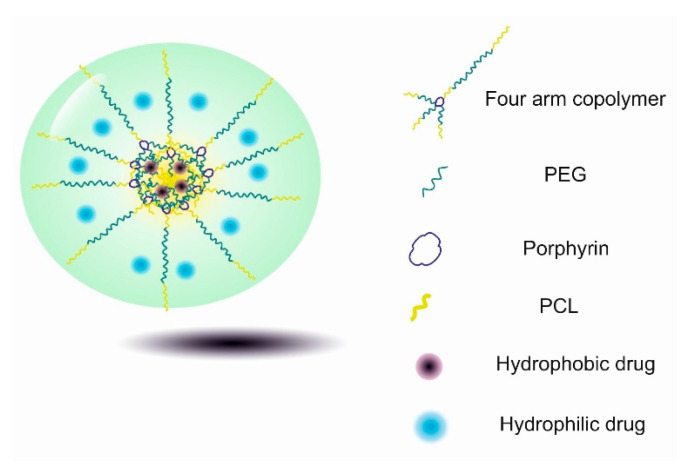
Thermosensitive fluorescent nanogels based on the four-arm PEG–PCL copolymer for co-delivery of hydrophobic and hydrophilic drugs.

Polyethylene glycol (PEG) is another important water-soluble, thermoresponsive polymer. The LCSTs of PEG-based polymers can be regulated by copolymerization with hydrophobic units [142]. Hydrophobic units like methyl and ethyl groups can regulate the polarity and temperature-responsiveness of the polymer within a physiological temperature range [143]. The composition of the monomers, molecular weight, concentration, and ionic strength of the solution considerably affect the LCST of PEG-based copolymers [144]. Xia Dong et al. synthesized a thermosensitive fluorescent nanogel using the four-arm PEG–PCL for bio-imaging applications. The results of the study demonstrated the superior capability of PEG–PCL as a drug carrier for tumour cells. Further, the PEG–PCL nanogels showed fluorescent activity in vivo while satisfying biocompatibility requirements, which made this nanogel system a suitable drug carrier for tumour-targeted delivery [138] (Figure 7).

### 3.3. Polymers Bearing Vinyl Ether Groups

#### 3.3.1. PMEO2MA

Poly (2-[2-methoxyethoxy] ethyl methacrylate) (PMEO2MA) is an amphiphilic and biocompatible polymer that contains PVE functional groups (O(CH=CH2)2). The phase transition behaviour of PMEO2MA is similar to PNIPAM, which enables this polymer to be used as a thermosensitive material in biomedical applications [145]. París et al. investigated the phase transition temperature of P(MEO2MA-co-DMAEMA) hydrogel. The copolymer was synthesized via free radical polymerization and the LCSTs of hydrogels with different contents of MEO2MA were investigated in PBS solution. The results demonstrated that the hydrogel was temperature and pH sensitive and LCST of the hydrogel could be tuned by changing MEO2MA content, ionic strength or the environment pH [146]. In another study, Shen et al. prepared a core–shell thermosensitive nanogel using reversible addition–fragmentation chain transfer polymerization based on PEG as the core and oligo (ethylene glycol) (OEG) as the outer layer of nanogels. MEO2MA was introduced as a thermoresponsive moiety. The synthesized nanogels with an average diameter of 40–80 nm had negligible cytotoxicity when tested on A549 cells [147].

**Figure 8:**
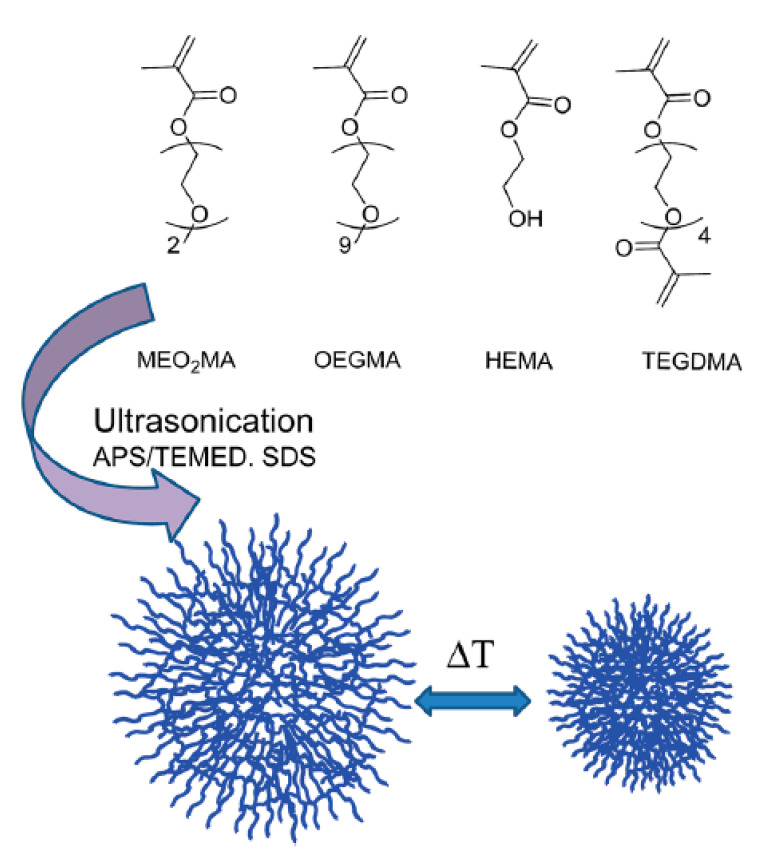
Thermosensitive nanocarriers based on MEO2MA and OEGMA with TEGDMA as the crosslinker. Adapted from Ref. [148] reproduced by permission of The Royal Society of Chemistry.

Biglione et al. synthesized a thermosensitive nanogel based on ethylene glycol using a facile ultra-sonication technique. Nanogels with 70 to 180 nm diameter were prepared using MEO2MA and oligo (ethylene glycol) methyl ether methacrylates (OEGMA) as temperature-responsive moieties and tetra ethylene glycol di-methacrylate (TEGDMA) as the crosslinker. Cytotoxicity and cell uptake evaluations were performed on A549 cells using RhB for labelling. The results indicate that the nanogels had appropriate cytotoxicity and cell permeation profiles [148] (Figure 8).

#### 3.3.2. OEGMA

Oligo (ethylene glycol) methyl ether methacrylate (OEGMA) has attracted attention as a new type of thermosensitive hydrogel [149]. Similar to PNIPAM, the LCST transition of OEGMA-based hydrogels is not very sensitive to external conditions. Therefore, ionic strength, concentration, and pH do not have a significant impact on the LCST transition of poly (OligoPOEGMA) [150]. Moreover, POEGMA polymers demonstrate exciting characteristics, including high anti-folding, nontoxicity, limited hysteresis, as well as adjustable temperature sensitivity [151]. Lutz et al. synthesized copolymers of MEO2MA and OEGMA via atom transfer radical polymerization and observed possible control of LCST between 26 and 90 °C by altering the monomer compositions [152]. Consequently, a large number of different types of polymers [153,154,155], micelles [156,157], vesicles [158], micro/nanogels [159], and smart POEGMA-based hydrogels [160] have been synthesized and investigated.

**Figure 9:**
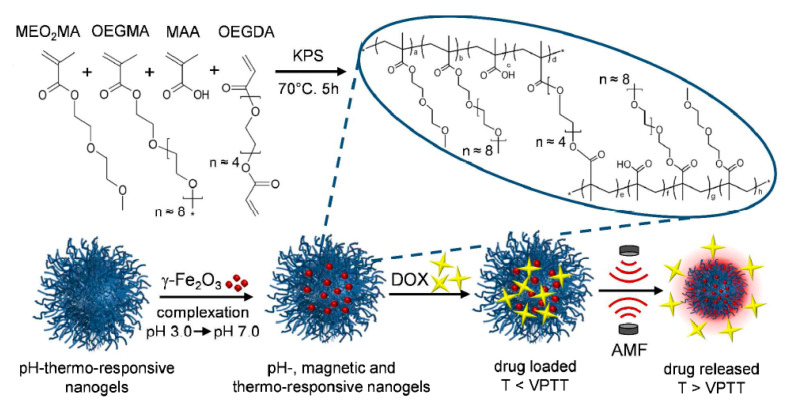
Temperature- and magnetic-field-responsive nanogels based on OEGMA for intracellular remote release of DOX. Adapted from Ref. [163] reproduced by permission of American Chemical Society.

OEGMA-based thermoresponsive materials are widely used as drug carrier hydrogels and nanogels [161,162]. Cortes et al. prepared a thermosensitive and magnetic-responsive nanogel for intracellular remote release of DOX. The prepared nanogels had a diameter from 320 to 460 nm, and their LCST was about 47 °C, which was appropriate for a thermal, magnetic hyperthermia strategy. It was also demonstrated that DOX release from the nanogels increased by the application of an alternating magnetic field [163] (Figure 9). When a high-frequency magnetic field is applied to magnetic nanoparticles (MNPs), they can generate heat, which is useful for hyperthermia treatment and acts as driving force for drug release [164]. The thermosensitive structures were used as chemical sensors and indicators. For instance, Liu and et al. synthesized OEG-based thermoresponsive, comb-like polymers via free radical polymerization, which was used as a temperature and pH-responsive sensor [165].

Previously studied thermosensitive nanogels with polymer-bearing vinyl ether groups are also shown in Table 2. Parameters such as size, interactions, thermosensitive part, therapeutic agent and application are all summarized.

### 3.4. Hydrophilic Polymers Bearing Hydrophobic Groups

The second approach to developing thermosensitive polymers is using hydrophobic moieties/polymers alongside hydrophilic materials. The most widely used hydrophilic materials are polysaccharides due to their biocompatibility. Different hydrophobic materials are used to conjugate on hydrophilic units to form thermosensitive materials. Many studies have been performed based on this approach, and different hydrophobic materials, such as cholesterol [184,185], poly L-lactide (PLLA) [186,187,188,189], beta glycerophosphate (β-GP) [190,191] and pluronic F127 (F127) [192,193] have been used to synthesize thermosensitive hydrogels and nanogels for biomedical applications.

#### 3.4.1. Cholesterol-Bearing Polymers

**Figure 10:**
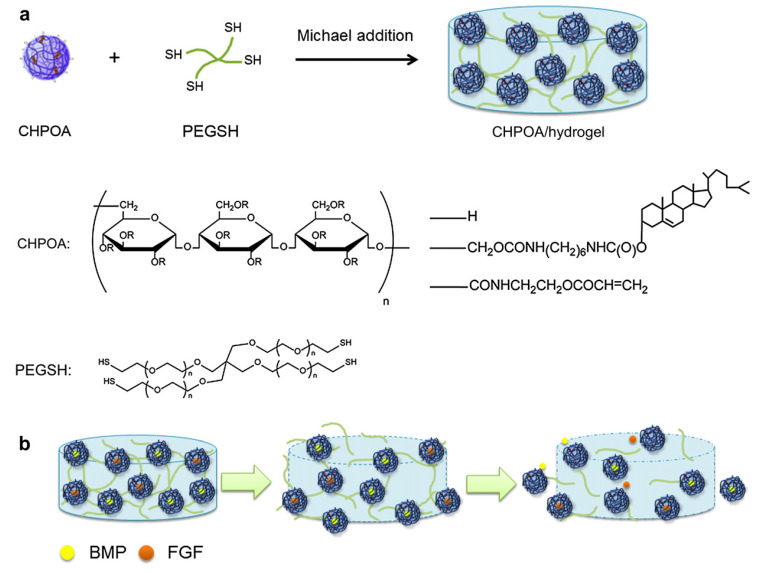
Cholesterol-bearing pullulan biodegradable nanogels for bone morphogenetic protein 2 (BMP2) and fibroblast growth factor 18 (FGF18) delivery for bone regeneration. (**a**) Synthesis of the acryloyl-group-bearing CHP (CHPOA)/hydrogel block and CHPOA nanogel and the chemical structure of pentaerythritol tetra (mercaptoethyl) polyoxyethylene (PEGSH). (‘R’ in CHPOA is H (glucose), cholesterol, and acryloyl). (**b**) Schematic diagram shows the release of FGF18 and BMP2 from cholesterol-bearing pullulan nanogels (green arrow represents the protein exchange reaction of serum albumin with FGF18 and BMP2). Adapted from Ref. [197] reproduced by permission of The Elsevier.

Cholesterol is an organic and hydrophobic molecule that exists in the mammalian body (component of the plasma membrane) and helps to make hormones, vitamin D, and compounds that aid in food digestion. In a few studies, cholesterol was used with pullulan (Plu) for nanogel formation and self-assembly of micelles [194,195,196]. Thara et al. prepared a self-assembled thermosensitive nanogel using cholesterol as a thermosensitive agent bearing hydroxypropyl cellulose (HP-Clu) with LCST of 50 °C. The diameter of nanogels was about 100 nm and 1500 nm at 37 °C and 60 °C in PBS, respectively [185]. In another study, Fujioka et al. synthesized cholesterol-bearing Plu nanogel for the delivery of bone morphogenetic protein-2 (BMP2) and Fibroblast growth factor-18 (FGF18) delivery. The results showed that the delivery of two proteins by the cholesterol-based nanogels aided in the regeneration of bone in vivo in a mouse model [197]. The protein exchange reaction, such as serum albumin with trapped BMP2 and FGF18 molecules, causes growth factor release over 8 weeks to maintain the BMP2 concentration at a certain level around the bone defects in the in-vivo condition. The schematic synthesis followed by growth factor delivery is depicted in Figure 10.

#### 3.4.2. PLLA-Bearing Polymers

Mechanical strength and the favourable degradation rate of aliphatic polyesters, such as PLLA, poly (lactide-glycolic acid) (PLGA), and PCL, make these polymers very attractive for the preparation of thermoresponsive nanogels. These hydrophobic polymers are mostly used in tissue engineering for constructing biodegradable scaffolds. However, mass transport of oxygen, nutrients and growth factors in such scaffolds is poor, and cell adhesion to the scaffold surface due to the hydrophobicity of polyesters is poor. Na et al. synthesized poly (l-lactic acid)/poly (ethylene glycol) and alternating multi-block temperature-responsive nanoparticles for anticancer drug delivery. The cytotoxicity of the nanoparticles was investigated with Lewis lung carcinoma (LLC) cells, and the results indicated that cell toxicity was temperature dependent and increased with increasing temperature from 37 °C to 42 °C [188]. Nagahama et al. prepared lysozyme-loaded Dex-g-PLLA nanogels and investigated the effect of the hydrophobic unit on the sustained release of lysozyme by comparing the release from Dex-g-PLLA conjugate with dextran-cholesterol. The synthesized Dex-g-PLLA nanogels had low critical aggregation concentrations. The results indicated that the Dex-g-PLLA nanogel has a high potential for protein delivery with sustained release of lysozyme for one week without denaturation [187]. Kyo et al. prepared Plu-g-PLLA nanogels with 150–800 nm diameters for sustained release of DOX. The PLLA in Plu-g-PLLA serves to induce self-assembly of nanogel and improves the loading of hydrophobic drugs [71]. In a recent study, Jung et al. developed Plu-g-PLLA nanogels with succinic anhydride (SA) to deliver lysozyme as a protein drug. The average diameters of the thermosensitive nanogels were 190 nm and 540 nm at 4 °C and 37 °C, respectively. In-vivo studies in nude mice indicated the sustained release of the drug [198].

#### 3.4.3. PLLA Bearing Polymers

Pluronic F127 is a hydrophilic non-ionic copolymer, based on non-toxic FDA-approved PEG and polypropylene glycol (PPG) segments, which is widely used in biomedical applications [192,199,200,201,202,203]. Sharma et al. developed a thermosensitive nanogel based on pluronic F127 as the carrier for the delivery of lidocaine (Lid) and prilocaine (Pl) and demonstrated by in-vitro and in-vivo experiments that the thermosensitivity of the carrier improves the delivery of Lid and PI [193]. Choi et al. prepared a nano-sponge based on heparin (Hep) and pluronic F127 and used it as a thermosensitive carrier for the controlled release of growth factors (bFGF, VEGF, BMP-2 and HGF) [204].

Previously studied thermosensitive nanogels with hydrophilic polymers bearing hydrophobic groups are shown in Table 3. Parameters such as size, interactions, thermosensitive part, therapeutic agent, and application are all summarized.

## 4. Conclusions

This review describes the synthesis and applications of thermoresponsive nanogels for targeted and controlled drug delivery. Thermoresponsive nanogels are discussed based on their thermally sensitive polymeric moieties. NIPAM is one of the most studied thermosensitive polymers that have been frequently used to prepare nanoparticles, hydrogels and nanogels for biomedical applications. However, non-biodegradability is limiting the use of NIPAM in clinical applications. Researchers are developing other thermoresponsive materials that are biodegradable for clinical applications while also possessing the rapid and sharp LCST of NIPAM. Materials that possess both hydrophobic and hydrophilic moieties in their molecular structure can induce nanogel formation and thermosensitivity. Based on the stated characteristics of thermosensitive nanogels, and from the authors’ perspective, thermosensitive polymers can be divided into four groups, including those bearing amide, ether and vinyl ether and hydrophilic polymers bearing hydrophobic groups. Generally, the above-mentioned thermosensitive polymers are conjugated to polysaccharides for augmenting biocompatibility as well as other desirable properties. As an alternative approach, hydrophilic polymers can be combined with hydrophobic materials such as PLLA and cholesterol to form thermosensitive nanogels. Four groups of thermosensitive materials were covered in this review, and some of the materials that are used in the synthesis of thermosensitive nanogels were presented. Multi-responsive nanogels, especially those with thermosensitive functionality, are commonly used in a vast number of biomedical applications, including cancer therapy, targeted delivery and in-situ gelation for drug release and entrapment; therefore, thermosensitive nanogels, with their invaluable functions, will become even more remarkable and important structures for drug delivery and tissue engineering applications in foreseeable future.

## Figures and Tables

**Table 1:** Components and applications of investigated thermosensitive nanogels containing polymers bearing amide groups. “-“ means there is no therapeutic agent introduced in the reference paper and only thermosensitive drug carrier was synthesized and prepared.

Component *	Thermosensitive Part	Size (nm)	Therapeutics	Application/Properties	Interactions	Ref.
P(NIPAM-AA)	NIPAM	125–325	-	heart repairing	hydrophobic and electrostatic	[106]
PTEGDA-b- P(NIPAM-co-NMA)	NIPPAM	300–480	DOX	thermo-responsive	hydrophilic	[107]
PEI-g-PNIPAM	NIPAM	200–350	plasmid gene P53	pH sensitive shell/temperature sensitive core	ionic	[108]
P(NIPAM-co- DMAEMA-co-AFA)	NIPAM	100	Cis	thermo-responsive	hydrophobic and hydrophilic	[109]
CS-NIPAM -MAA-	NIPAM	235	DOX	pH-/thermo-sensitive	electrostatic	[110]
starch-g -PNIPAM/ Fe3O4	NIPAM	67–79	MTX	magnetic and temperature responsive	hydrophobic and hydrophilic	[111]
NIPAM- (PAMAM)	NIPAM	200	Mall B	drug delivery system against cancer cells	hydrophobic and hydrophilic	[112]
(PNIPAM)	NIPAM	356	BSA	controlled protein delivery	hydrophobic and hydrophilic	[113]
PAMAM G3 –PNIPAM	NIPAM	200	5-Fu	enhancing 5-fluorouracil loading; cancer therapy	hydrophobic and hydrophilic	[114]
P(NIPPAM-AMPS)- TEGDMA	NIPAM	199–2211	DOX	pH-/thermo-sensitive	covalent	[115]
NIPAM- (dPG) -PANI	NIPAM	155–240	Anti-cancer drug	efficient in-vivo photothermal cancer therapy	hydrophobic and hydrophilic	[79]
mPEG-NIPAM- AA-MEA	NIPPAM	52–144	DOX	pH-/thermo-sensitive	electrostatic	[116]
PEDOT-NIPAM	NIPPAM	264	Cur	thermo-responsive	ionic	[117]
PNIPAM/(SA-GO)	NIPPAM	75–375	DOX	thermo-responsive	electrostatic	[118]
Alg-NIPAM	NIPPAM	180	DOX	redox-, pH- and thermo-sensitive	electrostatic	[119]
PNIPAM-g-PEI	NIPAM	300	Toxic protein Ricin A (RA) encoding plasmid DNA (pRA-EGFR)	thermo-responsive	hydrophobic and hydrophilic	[120]
(NIPAM-co-AA)	NIPAM	70–130	5-Fu	pH-/thermo-sensitive	hydrophobic and hydrophilic	[121]
P(NIPAM-NBD-SP)	NIPAM	90–130	-	thermo-responsive	covalent	[122]
NIPAM- PEDOT-PES	NIPPAM	195–295	DOX	thermo-responsive	hydrophobic and hydrophilic	[123]
NIPAM-AA-PEGDA	NIPAM	178–954	Mt	thermo-responsive	hydrophobic and hydrophilic	[124]
Salep-GO-NIPAM	NIPAM	93	Df and DOX	thermo-responsive	hydrophobic and hydrophilic	[125]
PDEAEMA-Fe3O4	PDEAAM	150–320	-	magnetic and thermo-sensitive	electrostatic	[66]
PDEAEMA - EGDMA	PDEAAM	160–360	-	pH-/thermo-sensitive	electrostatic	[126]
PAA- b-PDEAAM	PDEAAM	10–110	-	thermo-responsive	hydrophobic and hydrophilic	[92]
(DEA)/(DMA)	PDEAAM	165–288	-	thermo-responsive	hydrophobic and hydrophilic	[95]
(DEA)/(DMA)	PDEAAM	280–440	-	thermo-responsive	ionic	[93]
(PDEAAM)	PDEAAM	65–185	-	thermo-responsive	hydrophobic and hydrophilic	[127]
PEO-b-PDEAAM	PDEAAM	30–150	-	thermo-responsive	hydrophobic and hydrophilic	[94]
PDEAEMA	PDEAAM	200–800	Coumarin	thermo-responsive	covalent	[128]
PVCL-PAA	PVCL	175–300	Diclofenac	thermo-responsive		[129]
PVCL-Dex-MA	PVCL	100–400	-	thermo-responsive	hydrophobic	[130]
PVCL-PEGMA	PVCL	80–420	-	thermo-responsive	hydrophilic	[131]
Fib-g-PVCL	PVCL	150–170	5-Fu and Meg	thermo-responsive	ionic	[132]
PVCL	PVCL	140–280	-	nanogel with microfiber	hydrophobic and hydrophilic	[104]
PVCL-co-VFA and P(VP-co-VFA)	PVCL	70–180	-	pH-/thermo-sensitive	ionic	[133]
PDEAEMA/PVCL Dex-MA	PVCL	700–500	DOX	-		[77]
PVCL-AGA	PVCL	50–100	5-Fu	pH-/thermo-sensitive	hydrophobic and hydrophilic	[105]
PVCL-co-IA	PVCL	140–360	DOX	pH-/thermo-sensitive	hydrophobic and hydrophilic	[134]
P(VCL-co-AAPBA)	PVCL	120–250	Insulin	thermo-responsive	hydrophobic and hydrophilic and electrostatic	[135]
PVCL-PEGDA	PVCL	50–120	-	thermo-responsive	hydrophobic and hydrophilic	[103]
P(AETAC-X) - PNVCL	PVCL	155–770	-	thermo-responsive	ionic	[136]
P(ODGal- VCL-MAA)	PVCL	100–190	DOX	redox-, pH- and thermo-responsive	hydrophobic and hydrophilic	[137]
Por–PEG–PCL	PVCL	100–250	-	thermo-responsive	electrostatic attraction and hydrophobic interaction	[138]
POEOMA- b-PVCL	PVCL	150–920	NR as drug model	thermo-responsive	hydrophobic and hydrophilic	[139]
P(VCL/AAEMA /OEGMA)	PVCL	90–135	-	thermo-responsive	hydrophobic and hydrophilic	[140]
PDEGA-b- PDMA-b-PVCL	PVCL	20–400	-	thermo-responsive	covalent	[141]

* Full names of abbreviations are available in Appendix A.

**Table 2:** Components and applications of investigated thermosensitive nanogels containing polymers bearing vinyl ether groups. ”-“means there is no therapeutic agent introduced in the reference paper and only thermosensitive drug carrier was synthesized and prepared.

Component *	Thermosensitive Part	Size (nm)	Therapeutics	Application/Properties	Interactions	Ref.
ZnO-Au @PEG	PEG	15–57	TZ	thermo-responsive	hydrophobic and hydrophilic	[166]
(PEG-b-PADMO)	PEG	10–80	-	thermo-responsive	covalent	[142]
P(PEG-CPP-SA)	PEG	80–215	DOX	thermo-responsive	hydrophobic and hydrophilic	[167]
PEG-PPG-PEG	PEG	12*322	-	thermo-responsive	hydrophobic and hydrophilic	[168]
PEEP-PEG-PEEP	PEG	150–650	DOX	thermo-responsive	hydrophobic and hydrophilic	[169]
PEG-PLL-PLA-HA	PEG	160–220	BSA	thermo-responsive	hydrophobic and hydrophilic	[170]
P(MEA-co-PEGMEA)	PEGMEA	28–100		thermo-responsive	ionic	[171]
LAEMA-b-(PEGMA-co-LAEMA)	PEGMA	34–315	Pt, BSA, BG	thermo-responsive	hydrophobic and hydrophilic	[172]
PEGMA-CVP	PEGMA	85–205	RhB	thermo-responsive	ionic	[173]
PEGMA- Maleimide-dithiol	PEGMA	10–192	-	thermo-responsive	hydrophilic	[174]
Hg NPs@ P(MEO2MA -co-OEGMA)	MEO2MA, OEGMA	65	Bupivacaine	thermo-responsive	covalent and electrostatic	[161]
MEO2MA-PEGMA	MEO2MA	40–80	-	thermo-responsive	hydrophilic	[147]
DMDEA- OEGMA-BADS	OEGMA	17–58	Paclitaxel, DOX	thermo-responsive	hydrophobic	[159]
P[(LAEMA-MA)-b -(DEGMA-MBAM-LAEMA)]	DEGMA	60–180	IAZA	thermo-responsive	hydrophobic	[175]
MEO2MA – OEGMA-HEMA	MEO2MA, OEGMA	71–180	RhB as label	thermo-responsive	covalent	[148]
MEO2MA-OEGMA	MEO2MA, OEGMA	45	DOX	thermo-responsive	hydrophobic and hydrophilic	[176]
PCL-b-P(MEO2MA-co-OEGMA) Mn-Zn-Fe2O4	MEO2MA, OEGMA	33–129	DOX	temperature and magnetic responsive	hydrophobic and hydrophilic	[177]
Clay/ P(MEO2MA -co- POEGMA)	MEO2MA, OEGMA	200–400	-	nanogel/hydrogel nanocomposite	hydrophobic and hydrophilic	[160]
MEO2MA-ChS @Carbon QDs	MEO2MA	125–350	DOX	pH-/thermo-sensitive	electrostatic	[26]
CMC-MEO2MA-OEOMA-DMA	MEO2MA	10	DOX	pH-/thermo-sensitive	electrostatic	[178]
Ag-Au @ MEO2MA-HA	MEO2MA	10*60	TZ	HA as targeting, bimetallic NP as imaging	hydrophobic and hydrophilic	[179]
QDs-SEMA- PMEO2MA	MEO2MA	6	-	smart luminescent	hydrophobic and hydrophilic	[180]
Ag/Au @PS- MEO2MA-co- MEO5MA	MEO2MA	20–40	Cur	thermo-responsive	hydrophobic and hydrophilic	[181]
P(MEO2MA-co-OEGMA-co-MAA)	OEGMA	260–650	DOX	temperature and magnetic sensitive	hydrophobic and hydrophilic and electrostatic	[163]
dPG-OEGMA-DEGMA	OEGMA	50–200	-	thermo-responsive	hydrophobic and hydrophilic	[182]
HA-P(DEGMA-co-OEGMA)	OEGMA	150–214	hydrophobic dye	thermo-responsive	hydrophobic and hydrophilic	[183]
P(MEODEGM- AEMA-MPC)	MEODEGM	45–282	insulin	thermo-responsive	Ionic and electrostatic	[54]

* Full names of abbreviations are available in Appendix A.

**Table 3:** Components and applications of investigated thermosensitive nanogels containing hydrophilic polymers bearing hydrophobic groups. ”-“means there is no therapeutic agent introduced in the reference paper and only thermosensitive drug carrier was synthesized and prepared.

Component *	Thermosensitive Part	Size (nm)	Therapeutics	Application/Properties	Interactions	Ref.
POP-PS	POP	250–600	hGH	thermo-responsive	ionic and hydrophobic and hydrophilic	[205]
cholesterol bearing HP-Clu	Chl	29–82	-	thermo-responsive	hydrophobic and hydrophilic	[185]
PLLA-ChS Nisin	PLLA	180–300	nisin	target delivery for infection disease	esterification	[206]
Succinylated pullulan -g- PLLA	PLLA	190–520	lysozyme	thermo-responsive	electrostatic and hydrophobic interactions	[198]
S-Plu-g-OLLA	PLLA	250–450	amino acids	thermo-responsive	electrostatic	[207]
Plu-g-PLLA	PLLA	202–341	DOX	thermo-responsive	hydrophobic and hydrophilic	[71]
Fe3O4@mSiO2-PEO-PLA	PLLA	85–150	DOX	thermo-responsive	esterification	[208]
Plu-g- PLLA	PLLA	120–160	DOX	thermo-responsive	hydrophobic	[209]
F-127 and Hep	F-127	50–525	bFGF, HGF VEGF, BMP-2,	thermo-responsive	ionic	[204]
ChS - β-GP	β-GP	100–500	ethosuximide	thermo-responsive	hydrophobic	[210]
P(GME-co-EGE)	P(GME-co-EGE)	110–160	-	thermo-responsive	hydrophobic	[211]
F-127 and T80	F-127	32.5	Lid and Pl	thermo-responsive	hydrophobic and hydrophilic	[193]
F-127 and Hep	F-127	133	Cis	thermo-responsive	hydrophobic and hydrophilic	[212]
PEO-PPO- PEO	PPO	60–360	Mc	thermo-responsive	hydrophobic and hydrophilic	[213]
Bi2O3 @PVA	PVA	80–185	TZ	thermo-responsive	hydrophobic and hydrophilic and covalent	[214]
Gel A-GA	Gel A	60–250	-	thermo-responsive	-	[215]
HP-Clu and PMMA	HP-Clu	150–240	-	pH-/thermo-sensitive	hydrophobic and hydrophilic	[216]
HP-Clu- (PIA-co-PMA)	HP-Clu	100–610	DOX	pH-/thermo-sensitive	electrostatic	[217]
NAGA -DAAM	NAGA	50–600	-	thermo-responsive	hydrophobic and hydrophilic	[218]
P(L-Asp-co- PEG)- capryl	caprylic acid	7–180	-	thermo-responsive	hydrophobic interaction	[219]

* Full names of abbreviations are available in Appendix A.

## Appendix A

**Table A1:** Abbreviations.

Component	Abbreviation	Component	Abbreviation
(2-acetoacetoxyethyl) methacrylate	AAEMA	1,3-bis(carboxyphenoxy) propane	CPP
1-vinylimidazole	Vim	2-(2-methoxyethoxy) ethyl meth acrylate)	MeO2MA
2-(5,5-dimethyl-1,3-dioxan-2-yloxy) ethyl acrylate	DMDEA	2-(acetylthio) ethyl methacrylate	AcSEMA
2,2-bis(2-oxazoline)	BOX	2-aminoethyl methacrylamide hydrochloride	AEMA
2-dimethyl(aminoethyl) methacrylate	DMAEM	2-dimethylmaleinimido ethylacrylamide	DMIAAm
2-hydroxyethyl methacrylate	HEMA	2-lactobionamidoethyl methacrylamide	LAEMAm
2-methacryloyloxyethyl acrylate	MEA	2-methoxyethyl acrylate	MEA
3-(trimethoxysilyl)propyl methacrylate)	MPMA	3-{[(2R)-2-(octadecylamino)-3-phenyl propanoyl]amino}butyrate	TEAB
3-acrylamidophenylboronic acid	AAPBA	3-gluconamidopropyl methacrylamide	GAPMA
4-(2-acryloylaminoethylamino)-7-nitro-2,1,3-benzoxadiazole	NBDAA	4-acrylamidofluorescein	AFA
5-fluorouracil	5-Fu	6-O-vinyladipoyl-D-galactose	ODGal
Acetamidophenol	AP	Acrylamide	AAM
Acrylamidoglycolic acid	AGA	Acrylic acid	AA
Alginate	Alg	Atom transfer radical polymerization	ATRP
Basic fibroblast growth factor	bFGF	Beta glactosidase	BG
Beta glycerophosphate	β-GP	Bevacizumab	Bz
Bis (2-acryloyloxyethyl) disulfide	BADS	Bisacrylamide	BAM
Bismuth (III) oxide	Bi2O3	Bone morphogenetic protein 2	BMP-2
Bovine serum albumin	BSA	Bupivacaine	BV
Butyl methacrylate	BMA	Butyl methylacrylate	PIB
Butyl methylacrylate	BMA	Carboxymethyl cellulose sodium salts	CMC
Carboxymethyl hexanoyl chitosan	CHChS	Cellulose	Clu
Chitosan	CHS	Cholesterol	Chol
Chondroitin sulfate	CS	Cisplatin	CIS
Cloud point temperature	CPT	Coumarin 102	C 102
Covinyl pyrrolidone	CVP	Curcumin	Cur
Cytochrome C	Cyt C	Dendritic polyglycerol	dPG
Deoxyribonucleic acid-acrylamide	DNA-AAM	Dextran	Dex
Dextran methacrylates	Dex-MA	Di(ethylene glycol) methyl ethyl methacrylate	DEGMA
Diacetone acrylamide	DAAM	Dibucaine	Dc
Diclofenac	Df	Dimethyl maleinimido acrylamide	DMIAAM
Doxorubicin	Dox	Ethosuximide	ESM
Ethylene glycol dimethacrylate	EGDMA	Ethylene glycol dimethacrylate	EGDMA
Ethylene glycole	EG	Fibrinogen	Fib
Fibroblast growth factor 18	FGF18	Food and Drug Administration	FDA
Gelatin type A	Gel A	Glutaraldehyde	GA
Graphene oxide	GO	Heparin	Hep
Hollow gold nanoparticle	HGNP	Human growth hormone	hGH
Human serum albumin	HAS	Hyaluronic acid	HA
Hydroxymethyl acrylamide	HMAA	Hydroxypropyl cellulose	HP-Clu
Indomethacin	Imc	Iodoazomycin Arabinofuranoside	IAZA
Itaconic acid	IA	Lewis lung carcinoma	LLC
Lidocaine	Lid	Lower critical aggregation concentration	LCAC
Lower critical solution temperature	LCST	L-Proline	L-Pro
Maleic acid	MA	Maleimide dithiol	MDT
Malloapelta B	Mall B	Megestrol acetate	Meg
Melatonin	Mt	Mesoporous silica	mSiO2
Methacrylate	MA	Methacrylic Acid	MAA
Methotrexate	MTX	Methoxy-poly(ethylene glycol)	Met-PEG
Monomethoxy poly(ethylene glycol)	mPEG	Muscone	Mc
N, N-di ethylacrylamide	DEAAM	N,N′-methylenebis(acrylamide)	MBAM
N,N-diethylacrylamide	DEA	N,N-dimethylacrylamide	DMA
N,N-dimethylaminoethyl methacrylate	DMAEMA	N-acryloyl-3-aminophenylboronic acid	APBA
N-acryloylglycinamide	NAGA	Naphthalimide-based dye	NPTUA
Nile red	NR	Nitrobenzoxadiazole	NDB
Nitrobenzoxadiazole	NBD	N-methylolacrylamide	NMA
N-tert-butyl acrylamide	NTBA	N-vinylcaprolactam	NVCL
N-vinylformamide	VFA	N-vinylpyrrolidone	VP
Oligo (ethylene glycol)	OEG	Oligo (ethylene glycol) methacrylates	OEGMA
Oligo (ethylene glycol)methyl ether methacrylate	MEO5MA	Oligo (ethylene oxide) monomethyl ether methacrylate	OEOMA
Oligo (L-lactide)	OLA	Paclitaxel	PTX
Phenylboronic acid	PBA	Phenylethynesulfonamide	PES
Phosphate-buffered saline	PBS	Photochromic spiropyran	SP
Pluronic F127	F127	Poloxamer 407	P407
Poly (2-(2-methoxyethoxy) ethyl meth acrylate))	PMEO2MA	Poly (2-(2-methoxyethoxy)ethyl methacrylate)	PMEO2MA
Poly (2-(diethylamino)ethyl) methacrylate	PDEAEMA	Poly (2-aminoethyl methacrylamide hydrochloride)	PAEMA
Poly (2-Ethoxy-2-oxo-1,3,2-dioxaphospholane)	PEEP	Poly (2-isopropyl-2-oxazoline)	piPOz
Poly (2-methacryloyloxyethyl phosphorylcholine)	PMPC	poly (2-methoxyethyl acrylate)	PMEA
Poly (2-methylthioethyl glycidyl ether)	PMTEGE	Poly (3,4-ethylenedioxythiophene)	PEDOT
Poly (acrylamide)	PAM	Poly (acrylonitrile)	PAN
Poly (amino carbonate urethane)	PACU	Poly (caprolactone)	PCL
Poly (ether)	PE	Poly (ethyl glycidyl ether)	PEGE
Poly (ethylene glycol dimethacrylate)	PEGDMA	Poly (ethylene glycol) diacrylate	PEGDA
Poly (ethylene glycol) methacrylates	PEGMA	Poly (ethylene glycol) methyl ether acrylate	PEGMEA
Poly (ethylene glycole)	PEG	Poly (ethylene oxide)	PEO
Poly (glycidol)	PGL	Poly (glycidyl methyl ether)	PGME
Poly (lactide co-glycoside)	PLLA-co-GS	Poly (lactide-glycolic acid)	PLGA
Poly (L-alanine)	PLA	Poly (L-aspartic acid)	P(L-Asp)
Poly (L-lactide)	PLLA	Poly (L-lysine)	PLL
Poly (methacrylic acid)	PMA	Poly (methoxydiethylene glycol methacrylate)	PMEODEGM
Poly (methyl glycidyl ether)	PGME	Poly (N, N-di ethylacrylamide)	PDEAAM
Poly (N,N-dimethylacrylamide)	PDMA	Poly (N-acryloyl-2,2-dimethyl-1,3-oxazolidine	PADMO
Poly (N-isopropyl meth acryl amide)	PNIPMAM	Poly (N-isopropylacrylamide)	PNIPAM
Poly (N-n-propylacrylamide)	PNNPAM	Poly (N-vinyl caprolactam)	PVCL
Poly (oligo (ethylene glycol) methacrylates)	POEGMA	Poly (organo phosphazene)	POP
Poly (phenylboronate ester) acrylate	PPBDEMA	Poly (propylene oxide)	PPO
Poly (sodium 2-acrylamido-2-methylpropanesulfonate)	PAMPS	Poly (sodium styrenesulfonate)	PSSNa
Poly (urethane)	PU	Poly (vinyl ether)	PVE
Poly [(3-acrylamidopropyl)-trimethylammonium chloride]	PAMPTMA	Poly acrylamide	PAA
Poly ethylenimine	PEI	Poly Oligo (ethylene oxide) methyl ether methacrylate	POEOMA
Poly propylene glycol	PPG	Poly tetra (ethylene glycol) diacrylate	PTEGDA
Polyacrylic acid	PAA	Polyamidoamine	PAMAM
Polyaniline	PANI	Polyglycerol	PG
Polyvinyl alcohol	PVA	Porphyrin	Por
Prednisone	Pn	Prilocaine	Pl
Propranolol	Ppl	Propyl acrylic acid	PAA
Protamine	Pt	Protamine sulfate	PS
Pullulan	Plu	Quantum dots	QDs
Rhodamine B	RhB	Ricin A	RA
Salicylic acid	SCA	Sebacic acid	SA
Sodium 2-acrylamido-2-methylpropane sulfonate	AMPS	Sodium alginate	SA
spiropyran	SP	Styrene	ST
Succinic anhydride	SA	Succinylated pullulan	S-Plu
Temozolomide	TZ	Tetraethylene glycol dimethacrylate	TEGDMA
Tween 80	T80	Vascular endothelial growth factor	VEGF
